# Lack of knowledge about chronic kidney disease and its consequences

**DOI:** 10.1590/2175-8239-JBN-2023-E004en

**Published:** 2023-06-09

**Authors:** Marcus Vinícius de Pádua, Gustavo Navarro Betônico

**Affiliations:** 1 Universidade Federal de Uberlândia, Uberlândia, MG, Brazil.; 2 Faculdade de Medicina de Assis, Assis, SP, Brazil.

Chronic kidney disease (CKD) is a global health problem that is more severe in developing countries, mainly due to limited resources^1^. It is defined as a glomerular filtration rate (GFR) <60 mL/min/1.73 m^2^ or kidney damage or the association of both for a period of at least 3 months^2^.

However, almost all measures that help prevent disease progression and reduce associated complications, such as lifestyle modifications, medication adherence, maintenance of optimal blood glucose and blood pressure (BP) levels^3^, avoidance of further nephrotoxic insults^4^, and in advanced stages, maintenance of strict diet control, are heavily dependent on patient self-management^5^.

Knowing how each action affects an individual’s health, how much some measures can influence the evolution of some pathologies, particularly when it involves changing habits and lifestyle, is a prerequisite for this behavior change.

In recent decades, several genetic, environmental, sociodemographic, and clinical factors have been associated with CKD. In high-income countries, the sociodemographic factor plays a well-defined role in the epidemiology of CKD, as it is known that racial or ethnic minorities and people with lower socioeconomic status have a high incidence and prevalence of chronic non-communicable diseases^6^.

The association between socioeconomic level and risk of progressive CKD has also been described, so that recently the formula for calculating estimated glomerular filtration rate was modified to remove race/ethnicity from the calculation because it is more related to social construction rather than biological^7^.

In the medical literature, there is little information on how communication from health providers, whether primary care, specialists, or even non-specialized media, ultimately affects patients and their knowledge of kidney disease.

What is known is that the information does not reach at-risk groups, or if it does, it has a very low impact, perhaps due to the lack of specific communication from the provider on the subject^7^.

Another challenge is the population’s knowledge of the nephrology specialty, which was shown to be very low in a study carried out in Niteroi, Rio de Janeiro, Brazil^8^, reinforcing the already existing understanding of this knowledge also needs to be disseminated to the population.

There are studies that examine audio recordings between primary care teams and patients at risk of CKD, and these demonstrate that the discussion rarely focuses on the topic of kidney disease. When effective communication takes place, there is a significant increase in the patient’s knowledge about the risks of abuse of nephrotoxic medications and the benefits of CKD treatment, including the choice of dialysis modality that may be necessary^7^.

Structured education is fundamental to engage people with diabetes and other risk factors for CKD in the care of their disease and, consequently, in the necessary participation in shared decisions regarding the treatment plan. We live in a time of important discoveries and exciting changes in the management of CKD with better understanding of its pathogenesis, diagnoses, and therapies^2^. Collaborative, multidisciplinary, ongoing, and sustained efforts by all stakeholders are critical to support initiatives to improve the health and outcomes of the CKD population^2^.

The study by Albuquerque et al.^9^ adds another important finding to this scenario, despite some limitations reported by the authors, indicating that the population’s level of knowledge about CKD is generally very low, and even more so when adjusted for educational and socioeconomic levels. Obtaining data on the general public’s knowledge of CKD, preferably in different populations and regions of Brazil, is essential for designing health education programs targeting kidney disease. Without understanding the knowledge gaps in the population, public health education programs cannot be strategically planned and thus have very limited benefits.

Education and dissemination of knowledge on CKD should involve nephrologists and non-specialists alike. Strategies such as the inclusion of estimated Glomerular Filtration Rate (eGFR) in serum creatinine levels have increased awareness of CKD and referrals to nephrologists, but it is important to emphasize that eGFR alone is not sufficient for clinical decision making.

Another important point that draws attention in the study by Albuquerque et al.^9^ is that the family members of these patients with CKD had a greater knowledge about CKD^8^, perhaps due to the greater curiosity that is aroused in them, as is the case in several other pathologies that also become known through various campaigns, sometimes associated with months and colors, aimed at raising awareness among the entire population.

Finally, the study’s message that “new health education strategies are necessary, in order to obtain greater efficiency in the prevention of the disease” is of great relevance given the great growth in the global prevalence of CKD, whether developed by the Brazilian Society of Nephrology with the participation of its regional offices or by the Ministry of Health.
